# Retrotransposon Proliferation Coincident with the Evolution of Dioecy in *Asparagus*

**DOI:** 10.1534/g3.116.030239

**Published:** 2016-06-23

**Authors:** Alex Harkess, Francesco Mercati, Loredana Abbate, Michael McKain, J. Chris Pires, Tea Sala, Francesco Sunseri, Agostino Falavigna, Jim Leebens-Mack

**Affiliations:** * Department of Plant Biology, University of Georgia, Athens, Georgia 30602; † Donald Danforth Plant Science Center, St. Louis, Missouri 63132; ‡ Dipartimento di AGRARIA, Università Mediterranea di Reggio Calabria, Salita Melissari, 89124 Reggio Calabria, Italy; § Institute of Biosciences and BioResources, Division of Palermo, National Research Council, 900129 Palermo, Italy; ** Division of Biological Sciences, University of Missouri, Columbia, Missouri 65211; †† Consiglio per la Ricerca in Agricoltura e l’analisi dell’economia agraria (CREA), Research Unit for Vegetable Crops, Montanaso Lombardo, 26836 Lodi, Italy

**Keywords:** *Asparagus*, dioecy, sex chromosomes, transposons, genome size

## Abstract

Current phylogenetic sampling reveals that dioecy and an XY sex chromosome pair evolved once, or possibly twice, in the genus *Asparagus*. Although there appear to be some lineage-specific polyploidization events, the base chromosome number of 2*n* = 2× = 20 is relatively conserved across the *Asparagus* genus. Regardless, dioecious species tend to have larger genomes than hermaphroditic species. Here, we test whether this genome size expansion in dioecious species is related to a polyploidization and subsequent chromosome fusion, or to retrotransposon proliferation in dioecious species. We first estimate genome sizes, or use published values, for four hermaphrodites and four dioecious species distributed across the phylogeny, and show that dioecious species typically have larger genomes than hermaphroditic species. Utilizing a phylogenomic approach, we find no evidence for ancient polyploidization contributing to increased genome sizes of sampled dioecious species. We do find support for an ancient whole genome duplication (WGD) event predating the diversification of the *Asparagus* genus. Repetitive DNA content of the four hermaphroditic and four dioecious species was characterized based on randomly sampled whole genome shotgun sequencing, and common elements were annotated. Across our broad phylogenetic sampling, *Ty-1 Copia* retroelements, in particular, have undergone a marked proliferation in dioecious species. In the absence of a detectable WGD event, retrotransposon proliferation is the most likely explanation for the precipitous increase in genome size in dioecious *Asparagus* species.

Fewer than 10% of flowering plant species are dioecious—the condition where individual plants are distinctly male or female (Ainsworth 2000). Gender in some dioecious plants can be governed by a sex chromosome pair, such as in papaya (*Carica papaya*), white campion (*Silene latifolia*), persimmon, *Rumex*, and garden asparagus (*Asparagus officinalis*) (Telgmann-Rauber *et al.* 2007; Ming *et al.* 2011; Akagi *et al.* 2014; Hough *et al.* 2014). The evolution of a distinct sex chromosome pair is hypothesized to be driven by the evolution of a nonrecombining region between the X and Y (or Z and W) chromosomes, where tightly linked sex determination genes reside (Charlesworth and Charlesworth 1978). Given the repeated and independent evolution of dioecy across the angiosperm phylogeny, the transition from autosome to sex chromosome is undoubtedly governed by different sex determination genes and evolutionary processes, and consequently must be viewed in a taxon-specific context. Despite this diversity in sex chromosome evolution across the angiosperms, two particularly interesting associations can be seen in some dioecious systems coincident with variation in sexual system: the proliferation of repetitive elements, and the occurrence of one or multiple whole genome duplication (WGD) (polyploidy) events.

As a consequence of restricted recombination between regions of sex chromosomes, repetitive elements tend to persist and replicate in an unbalanced way, accumulating preferentially on hemizygous regions of Y and W chromosomes. Transposable elements can be broadly classified primarily by their means of transposition (Wicker *et al.* 2007); class I retrotransposons move by a “copy and paste” mechanism, and replicate through an mRNA intermediate, which ultimately results in a net increase in the element’s copy number; whereas class II transposable elements move through a DNA intermediate in a “cut and paste” fashion. Since Class I retrotransposons can range in length from 5 kb to 20 kb or longer, their proliferation can lead to drastic and rapid changes in genome size (Kidwell 2002). This accumulation, especially of active retroelements, can be clearly seen when comparing the relatively young papaya X and hermaphrodite-specific region of the Y (HSY) (VanBuren and Ming 2013). Unbalanced accumulation of transposons and other repetitive elements, paired with the inability for recombination to remove them along with other deleterious mutations, is likely a major factor that leads to the initial physical expansion and genic degeneration of a young, partially nonrecombining Y or W chromosome (Steinemann and Steinemann 1998; Bachtrog *et al.* 2008; Bachtrog 2013). Transposons have also been directly implicated in the evolution of sex determination genes through disruption of gene expression. In melon (*Cucumis melo*), a class II hAT DNA transposon insertion is responsible for promoter hypermethylation and transcriptional repression of the zinc-finger transcription factor *CMWIP1*, heritably inducing the transition from monoecy to gynodioecy (Boualem *et al.* 2008).

An association between polyploidy and transitions in sexual system across the angiosperms is most clear in the *Fragaria* genus, where at least four independent WGD events have occurred across all major clades, leading to an abundance of polyploid dioecious species phylogenetically placed as sister to dioecious hermaphrodites (Rousseau-Gueutin *et al.* 2009; Ashman *et al.* 2013). However, loss of dioecy has also been associated with an increase in ploidy, as seen in one clade of *Mercurialis* (Krähenbühl *et al.* 2002). The mechanisms that potentially relate WGD events to the evolution of sexually dimorphic populations are variable and poorly understood, though again owing to the extreme complexity and species-specific nature of sex chromosome and dioecy.

Garden asparagus (*A. officinalis L*.) is a particularly useful dioecious plant for studying sex chromosome evolution given that it has cytologically homomorphic X and Y sex chromosomes, suggesting that the transition from hermaphroditism to dioecy was recent (Telgmann-Rauber *et al.* 2007; Kubota *et al.* 2012). Coincident with the evolution of dioecy was a range shift from South Africa into North Africa, Europe, and Asia (Štajner *et al.* 2002; Kubota *et al.* 2012; Norup *et al.* 2015). It was previously reported that dioecious *Asparagus* species tend to have larger genomes than hermaphrodites, but there was no evidence supporting a WGD event that separates the dioecious species from the hermaphrodites (Kuhl *et al.* 2005). The base chromosome number of 2*n* = 2× = 20 is generally consistent across the genus, except for instances of very recent polyploidization in some species (Kanno and Yokoyama 2011). These findings suggest one of two hypotheses may be responsible for an increase in genome size: one possibility is that a WGD occurred in the last common ancestor of all dioecious species, followed by a chromosome fusion or reduction; and another possibility is that repetitive DNA has proliferated to drive the increase in the genome sizes of dioecious species. Here, we test both hypotheses by first leveraging transcriptome assemblies for one hermaphroditic and one dioecious species to identify the relative timing of WGD events in the genus *Asparagus*. We then use shallow Roche 454 whole genome shotgun sequencing from four hermaphrodites and four dioecious species that are sampled from across the phylogeny to assess the repetitive element content of each species in relation to its genome size.

## Materials and Methods

### Flow cytometry genome size estimation

The genome sizes of *A. officinalis*, *A. virgatus*, and *A. asparagoides* were estimated by flow cytometry at the Benaroya Research Institute at Virginia Mason in Seattle, WA. Nuclei isolations from a single mature leaf were analyzed in three technical replicates for each species. The genome sizes of *A. aphyllus*, *A. stipularis*, and *A. falcatus* were estimated by flow cytometry using the known genome size of *A. officinalis* (1C-value = 1.37 pg) as a reference standard. Ten plants for each species, grown in a greenhouse, were sampled, and three randomly selected plants were analyzed. The analysis was carried out with the Partec PAS flow cytometer (Partec, http://www.partec.de/), equipped with a mercury lamp. Fully expanded leaves (0.1 g) were chopped in 300 μl nuclei extraction buffer (CyStain ultraviolet Precise P Nuclei Extraction Buffer; Partec, Münster, Germany) for 30–40 sec. The solution was filtered through a 30-mm Cell-Trics disposable filter (Partec), and 1.2 ml of staining solution containing 4,6-diamidino-2- phenylindole was added. The relative fluorescence intensity of stained nuclei was measured on a linear scale, and 4000–5000 nuclei for each sample were analyzed (Galbraith *et al.* 1998). DNA content histograms were generated using the Partec software package (FloMax). Given that the X and Y chromosomes in garden asparagus (*A. officinalis*) are cytologically homomorphic (Deng *et al.* 2012), representing a lack of degeneration and the relatively young age of Y, we did not discern between potential sex differences in the dioecious species.

### Transcriptome-based Ks analysis

Transcriptomes from dioecious *A. officinalis* and hermaphroditic *A. asparagoides* were used to infer a putative WGD event in the genus *Asparagus*. The transcriptome assembly and translation for *A. officinalis* was taken from Harkess *et al.* (2015) (http://datadryad.org/resource/doi:10.5061/dryad.92c60). We generated leaf RNA-Seq for *A. asparagoides* by first isolating total RNA from mature leaf tissue using a Qiagen RNeasy Plant Mini kit. Total RNA quantity and quality was assessed using an RNA Nano chip on the Bioanalyzer 2100. A sequencing library was generated using the TruSeq RNA Library Prep Kit v2 (Illumina) according to the manufacturer’s instructions, using 1 μg of total RNA input. The library was sequenced with paired-end 100-nt reads on an Illumina HiSeq2000, generating 55,686,513 read pairs (nearly 11 Gb of data). Reads were quality trimmed using Trimmomatic (v0.32), removing sequencing adapters, and clipping 3′ and 5′ read ends with a quality score lower than Phred 5. Cleaned reads were assembled using Trinity (r20140717) with default parameters. We filtered transcript isoforms with low support by removing isoforms with < 0.01% of the Trinity gene subcomponent read support. Coding sequence and peptide translations were inferred using TransDecoder (r20140704) with default settings. Raw sequence reads for *A. asparagoides* have been deposited under BioProject PRJNA326431.

Using a pipeline from McKain *et al.* (2012; https://github.com/mrmckain/FASTKs), we first identified putative paralogs in each filtered transcriptome assembly using all *vs.* all blastn (1e–40 cutoff). Peptide sequences for hit pairs longer than 100 amino acids were aligned using MUSCLE (v3.8.31), then codon alignments were inferred using PAL2NAL (v13) (Suyama *et al.* 2006). For each paralog pair, *Ks* was calculated using CodeML in PAML (Yang 2007) (v4.8).

### 454 pyrosequencing and transposon quantification

Whole genomic DNA was extracted from four hermaphroditic and four dioecious species using a CTAB method (Doyle and Doyle 1987). Sequencing libraries were prepared using the Roche 454 GS FLX Titanium library preparation kit according to the manufacturer’s instructions. Raw reads were first deduplicated to remove probable emulsion PCR sequencing artifacts, then filtered to remove reads < 100 nt long. Read names from all species were first prepended with a unique species identifier, and concatenated. The RepeatExplorer (v0.9.7.4) pipeline (http://www.repeatexplorer.org) was then used to cluster, assemble, and annotate all filtered shotgun reads against a custom garden *Asparagus* RepeatMasker database (see below) using otherwise default settings. Clustering and heatmap production of the 100 largest transposon clusters was performed using heatmap.2 in the gplot package in R (v3.2.1) using default settings; a distance matrix was generated using Euclidean distances, and hierarchical clustering was performed using “complete” clustering.

To improve the annotations of repetitive element clusters generated through the RepeatExplorer pipeline, instead of utilizing default RepeatMasker Viridplantae libraries, we generated a much higher coverage of 454 reads for *A. officinalis* to build a comprehensive database of annotated exemplar repeats for the *Asparagus* genus. A custom garden *Asparagus* RepeatMasker database was generated using similar methodology. A total of 893,623 454 FLX Titanium reads were generated from leaf tissue of a doubled haploid (YY) garden asparagus individual. Reads were more stringently filtered to a 150 nt minimum length. The same version of RepeatExplorer was then run, and the resulting cap3 consensus assemblies for each cluster were annotated using RepeatClassifier, part of the RepeatModeler (v1.0.8) suite, with default settings. A total of 22,361 sequences greater than 150nt in length and with annotations was retained for annotating all repetitive element clusters, and are available in Dryad (http://dx.doi.org/10.5061/dryad.1k450). Raw 454 shotgun sequence data for all individuals have also been deposited in Dryad.

### Data availability

The authors state that all data necessary for confirming the conclusions presented in the article are represented fully within the article.

## Results and Discussion

### Genome size increases in dioecious Asparagus

Genome sizes and ploidy vary greatly across the order Asparagales, with 1C values ranging from 0.3 pg to 88.2 pg (Leitch *et al.* 2010). Diploid dioecious *Asparagus* species have been reported as having genome sizes nearly double the size of diploid hermaphroditic congeners (Štajner *et al.* 2002; Fukuda *et al.* 2005; Kubota *et al.*, 2012). We first confirmed this by generating or supplementing published genome size estimations for eight *Asparagus* species, four hermaphrodites, and four dioecious species, sampled across all major clades of the *Asparagus* phylogeny (Kubota *et al.* 2012) (Table 1). All individuals have been documented as diploids (2*n* = 2× = 20) except for *A. maritimus*, a hexaploid (Štajner *et al.* 2002; Kanno and Yokoyama 2011). Flow cytometry-derived genome sizes (pg/1C) for hermaphrodites range from 0.72 to 1.06, while dioecious species range from 1.09 to 1.37. Dioecious species tend to have larger genome sizes than hermaphroditic species (unpaired *t*-test, *P* = 0.0173). An outlier is the hermaphrodite *A. asparagoides*, with a relatively large genome size (1C = 2.40; Dixon’s Q test, *P* = 0.074).

**Table 1:** Genome sizes, 454 pyrosequencing, and repetitive element clustering

Species	Sexual System	Picograms/Nucleus (Mean ± SD)	1C Value	Raw Reads	Filtered Reads	Clustered Reads (%)
*A. officinalis*	Dioecious	2.74 ± 0.044	1.37	29,677	26,525	54.4%
*A. maritimus*	Dioecious	7.87 ± 0.204*^a^*	1.31	49,616	45,036	53.7%
*A. aphyllus*	Dioecious	2.49 ± 0.007	1.25	47,322	42,808	58.9%
*A. stipularis*	Dioecious	2.17 ± 0.005	1.09	30,405	27,911	56.4%
*A. falcatus*	Hermaphrodite	2.11 ± 0.007	1.06	26,836	24,304	60.4%
*A. virgatus*	Hermaphrodite	1.66 ± 0.055	0.83	45,043	41,053	45.0%
*A. pyrimidalis*	Hermaphrodite	1.44 ± 0.037*^a^*	0.72	56,197	51,293	53.8%
*A. asparagoides*	Hermaphrodite	4.80 ± 0.062	2.40	41,952	37,435	59.2%
Sum				247,755	224,804	
Average				41,293	37,467	

a Data from Štajner *et al.* (2002).

### No evidence for a dioecy-specific polyploidy event

We employed a phylogenomics approach to test whether a WGD event separates the dioecious and hermaphroditic species in *Asparagus*. Transcriptome assemblies were generated for two species sampled broadly across the phylogeny: a basal diploid hermaphrodite (*A. asparagoides*; 2*n* = 2× = 20), and diploid dioecious garden asparagus (*A. officinalis*; 2*n* = 2× = 20). Intraspecific paralog pairs and interspecific orthologous gene pairs were inferred to generate *Ks* (synonymous substitution rate) distributions, and assess the relative timing of WGD event relative to speciation events (Blanc and Wolfe 2004; Cui *et al.* 2006; McKain *et al.* 2012; Doyle and Egan 2010). Despite being an outlier in terms of genome size, *A. asparagoides* was utilized for the comparison given that it is a basal member of the genus, shares the same diploid chromosome count as *A. officinalis*, and that transcriptome-based *Ks* analyses are independent of genome size.

**Figure 1:**
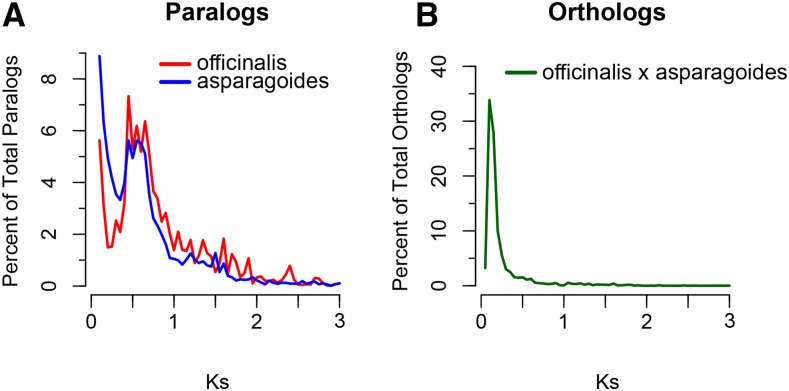
Transcriptome-based *Ks* frequency distributions for (A) paralogous, and (B) orthologous pairs of dioecious *A. officinalis* and hermaphroditic *A. asparagoides*. Paralogous and orthologous *Ks* distributions suggest a shared whole genome duplication event at *Ks* ∼0.5 that occurred before the diversification of the *Asparagus* genus.

Transcriptome assembly and translation results for the two species are presented in Supplemental Material, Table S1. One distinct, shared, polyploidization event (*Ks* ∼0.5) was inferred from the *Ks* frequency distribution of paralogous pairs in both *Asparagus* species (Figure 1 and Table S2). Additionally, orthologous pairs exhibit a *Ks* peak close to 0, representing low divergence, and suggestive of recent diversification of species and/or similar mutation rates. Comparison of orthologs and paralogs demonstrates that at least one detectable genome duplication event occurred before the diversification of the *Asparagus* genus (Figure 1).

A major limitation with *Ks* analyses is that more recent duplication events are difficult to detect (Blanc and Wolfe 2004; Cui *et al.* 2006). This issue is exacerbated when using *de novo* transcriptome assemblies, where recently duplicated paralogs can be computationally mistaken as alleles, and incorrectly collapsed into a single transcript sequence during the assembly process. Gene duplication, in addition to sequencing and assembly errors, can contribute to a high frequency of low *Ks* gene pairs. Given that the *A. officinalis* and *A. asparagoides* ortholog peak overlaps with the left-hand shoulder in the paralog *Ks* plot, the plots alone do not allow us to unambiguously reject recent WGDs within the *Asparagus* genus. Given that there are no current age estimates for the divergence of the *Asparagus* lineage, we cannot exclude the possibility that a more recent duplication event, such as one that may have co-occurred with the evolution of dioecy, could be undetectable by transcriptome data. However, such a whole genome duplication event would need to be followed by multiple chromosome fusion or loss events to reduce the chromosome number back to 2*n* = 2× = 20 found in most karyotyped dioecious *Asparagus* species (Kanno and Yokoyama 2011).

### Lineage-specific expansion of transposable elements

Given the lack of evidence that ancient polyploidy was responsible for the larger genome sizes of dioecious *Asparagus* species relative to hermaphroditic species, we assessed the alternative hypothesis that the genome size increases in dioecious species were at least partly due to transposon amplification. We utilized whole genome shotgun sequence reads to assess the repetitive content of hermaphrodite and dioecious *Asparagus* species using the RepeatExplorer Galaxy server (http://www.repeatexplorer.org). Briefly, this method utilizes all-by-all read comparisons followed by Louvain clustering (Blondel *et al.* 2008) to place reads into unbiased clusters of putative high copy elements, followed by a RepeatMasker annotation and cap3 assembly (Huang and Madan 1999).

A total of 327,048 raw reads were sequenced for the eight genomes using Roche 454 FLX chemistry, with genome coverages ranging from 0.0051× to 0.0234× (Table S3). After removing duplicate reads that were likely clonal, 321,865 total reads remained for analysis. To improve clustering, we then removed reads < 100 nt long, yielding a filtered set of 296,365 reads (mean = 37,047 reads per species) with a mean length of 321 nt. The filtered set of reads was concatenated and clustered using the RepeatExplorer pipeline, placing 162,435 reads into 29,643 repetitive element clusters (Table 1). Repetitive element clusters were filtered by read count, requiring at least 0.01% of the total filtered reads (30 reads), amounting to 336 clusters for downstream analysis. These clusters were annotated against a custom RepeatMasker database generated with additional data for dioecious *A. officinalis*. For a given cluster of repetitive elements, the repetitive fraction of each species’ genome was calculated as the number of a given species’ reads in a cluster, divided by the total number of reads sequenced for that species, represented as a percentage.

**Figure 2:**
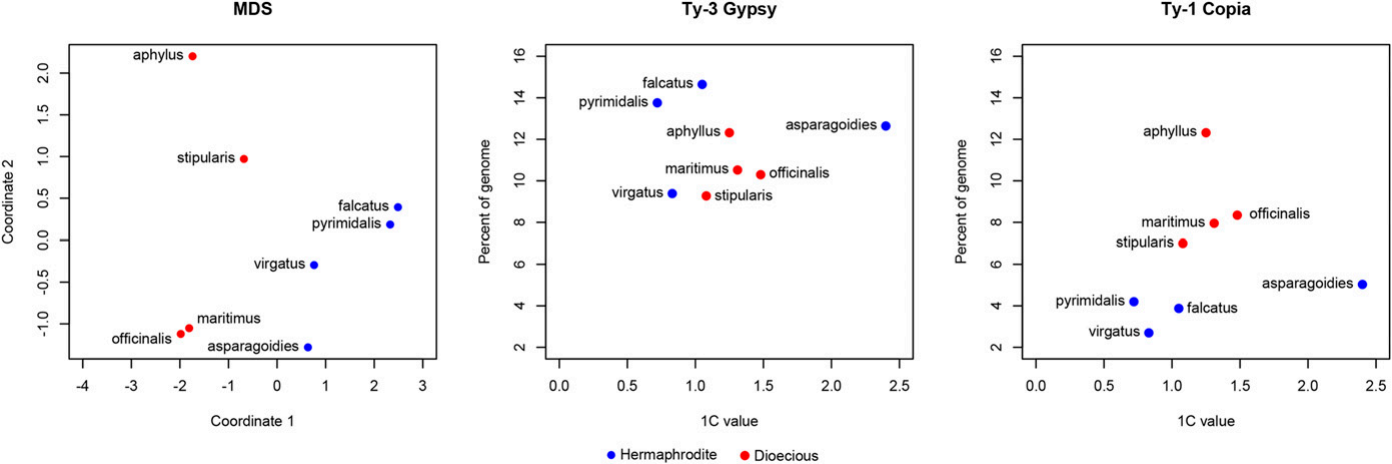
Multidimensional scaling (MDS) and relationship of genome size to *Gypsy* and *Copia* retroelement content for both dioecious and hermaphroditic genomes. Blue dots represent hermaphroditic species, while red dots represent dioecious species.

**Figure 3:**
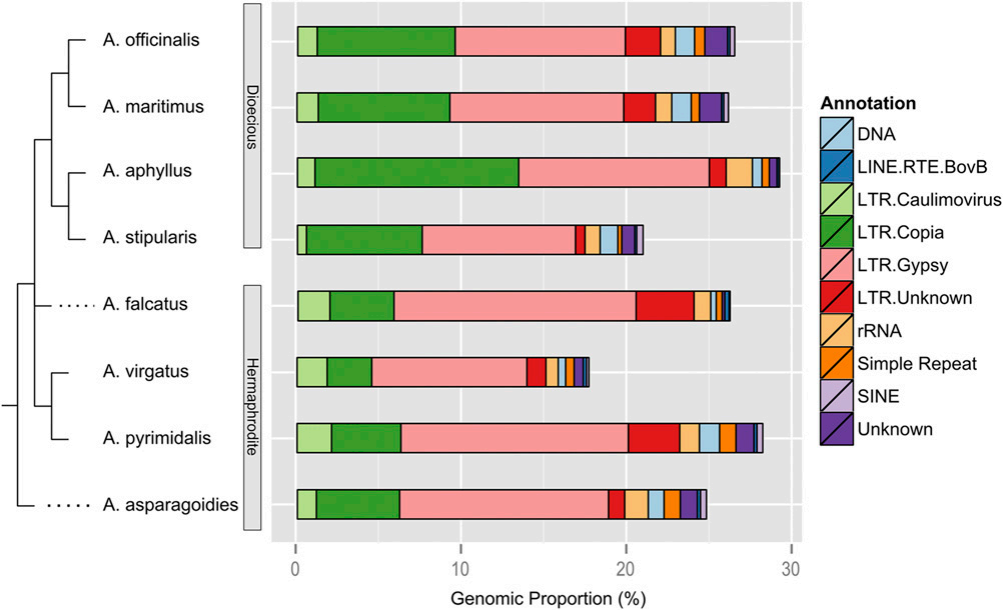
Cladogram of *Asparagus* species relationships with high copy repetitive elements. High copy elements refer to clusters with greater than 0.01% of the total read count in the multispecies analysis, able to be most confidently annotated against the custom *A. officinalis* repetitive element database. DNA transposons from several families were collapsed into a single annotation class.

Multidimensional scaling (MDS) analysis of the genomic proportions for all clusters shows that dioecious and hermaphroditic species form two distinct clusters (Figure 2). In general, *Gypsy* and *Copia* retrotransposons dominate the genomic landscape for all sampled *Asparagus* species (Figure 3). In all four dioecious species, *Gypsy* retrotransposons occupy a larger percentage of each genome than in the hermaphrodites, although *Copia* elements have distinctly expanded in the dioecious species (Figure 2). This suggests that both *Gypsy* and *Copia* elements increased in copy number in the dioecious species, and the proliferation of *Copia* elements was a more substantial contributor to the expansion of dioecious genome sizes. Taken together, the lack of evidence for recent WGDs described in Kuhl *et al.* (2005), paired with the increased abundance of *Copia* elements in dioecious species, supports our hypothesis that there are no recent WGDs in the *Asparagus* genus.

We identified 46 repetitive element clusters that were private to the dioecious species, and 37 clusters that were private to all hermaphroditic species. In the dioecious species, 26 clusters were *Gypsy*, and seven clusters were *Copia*, whereas in the hermaphroditic species, 12 clusters were *Gypsy*, and 11 clusters were *Copia*. This suggests that there is active turnover of transposable elements in the *Asparagus* genus, perhaps coincident with the evolution of dioecy and a sex chromosome. Additionally, it is possible that a small number of *Copia* elements may be largely responsible for the genome size expansion in dioecious species, but this would require whole genome assemblies and annotations as RepeatExplorer is limited in ability to finely delimit elements.

One caveat for performing a single repeat clustering analysis including all species (as opposed to individually analyzing each species) is that low frequency or moderately diverged sequences from phylogenetically distant species may not cluster. Additionally, there could be less power for detecting species-specific transposon family proliferations. Consequently, these estimates of repetitive element content are certainly underestimates of the total proportion of repetitive element content in each species’ genome. To understand the level of difference in these two analysis types, we generated 893,623 additional 454 shotgun reads (mean length 526 nt) for a mature double haploid YY *A. officinalis* individual, and ran the RepeatExplorer pipeline with this single species. The repeat content was estimated at 71.1%, much greater than the 54.4% that was estimated by concatenating eight species in a single analysis. This result suggests that the genomic proportions of transposons estimated through multispecies read clustering in this study should be interpreted as being underestimates, biased toward high copy elements with lower divergence between species, and used mostly for comparisons of high copy element percentages between species. The advantage of this analysis is that direct comparisons for a given transposon cluster can be assessed across all species, without the need to perform additional clustering between species. Without genome sequences and assemblies for several hermaphroditic and dioecious *Asparagus* species, including high quality repetitive element annotations and length distributions, we cannot quantify with certainty the nucleotide contribution that each transposable element class contributes to genome size increase.

The method of repeat quantification and sequence read type also largely affects the estimated proportion of repetitive elements. Repetitive element content has previously been estimated for *A. officinalis* in at least three separate studies. Vitte *et al.* (2013) directly annotated garden *Asparagus* Bacterial Artificial Chromosome (BAC) assemblies for transposon content. By comparing the sequence alignment identity of intact long terminal repeats (LTRs) from retroelements, and applying a clock estimation from rice retroelement divergence (Ma and Bennetzen 2004), Vitte *et al.* (2013) estimated that the majority of the *Asparagus* genome is comprised of young, recently inserted (< 6 million yr ago) and nested retroelements. Li *et al.* (2014) took a high-throughput sequencing approach, and inferred that the garden *Asparagus* genome is 53% repetitive by *de novo* assembling genomic paired-end 100 nt Illumina reads into a ∼400 Mbp assembly with a scaffold N50 of 1504 nt. Hertweck (2013) took a similar approach with 80-bp Illumina read data, and independently estimated 47% of the garden *Asparagus* genome as comprising repetitive elements. We hypothesize that our much higher estimation of 71.1% repetitive content is due largely to the increased detection power coming with longer 454 reads relative to 80–100 bp Illumina reads, and our use of RepeatExplorer’s unique assembly-free, graph-based clustering, and annotation of individual long reads.

### Transposon clustering yields phylogenetic signal

**Figure 4:**
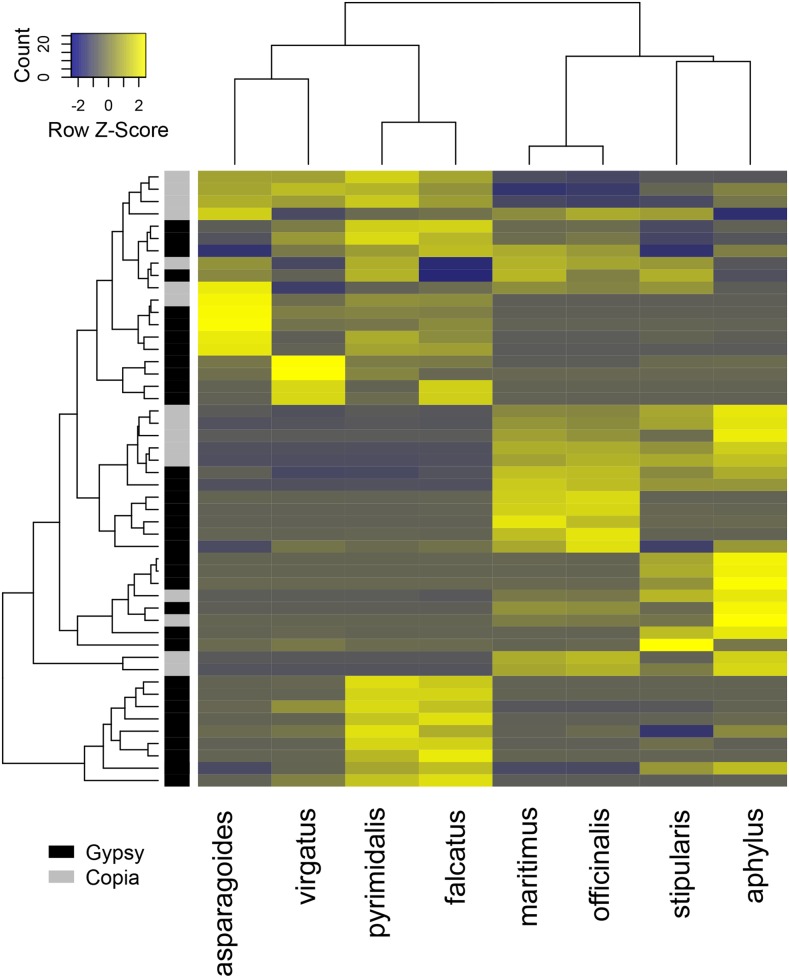
Heatmap clustering of 100 largest *Gypsy* or *Copia* element clusters. Rows represent individual clusters, annotated as *Gypsy* (black), and *Copia* (gray).

Clustering of the genomic proportions for the 100 largest *Gypsy* and *Copia* retrotransposon clusters also reveals phylogenetic signal in the data (Figure 4). The deepest branch divides the hermaphroditic and dioecious species from each other, and all species are paired with their closest phylogenetic neighbor given the current phylogeny and sampling from Kubota *et al.* (2012), with the exception of the earliest diverging species in the genus, *A. asparagoides*. The genomic proportions of repetitive elements have been used to identify phylogenetic signal in several plant species with species relationships that have been difficult to resolve with traditional low copy gene sequencing (Dodsworth *et al.* 2014). While our clustering approach may be less able to detect low- and medium-frequency repeats compared to the approach of Dodsworth *et al.* (2014), here we show a complementary analysis that yields similar results using high copy transposon clusters.

Recently, Norup *et al.* (2015) proposed two origins of dioecy within *Asparagus*, providing an alternative to the previously hypothesized single origin (Kubota *et al.* 2012). Our sampling includes species derived from both of the hypothesized origins of dioecy from Norup *et al.* (2015), which indicates that dioecy evolved in one clade that includes *A. officinalis* and *A. maritimus*, as well as another clade that includes *A. stipularis* and *A. aphyllus*. In the case of multiple origins of dioecy, without hermaphroditic outgroup species for each origin, our limited sampling does not allow us to describe the potentially different repetitive element radiations in the two dioecious clades. Further, it is possible that transposon proliferation and genome size increase occurred in the common ancestor of both dioecious lineages, predating the origin of dioecy. Rigorous testing of a general relationship between transposon activity and the origin of sex chromosomes will come with future meta-analyses including data from this and other comparative studies of transposon activity in hermaphrodite and dioecious lineages.

Compared to *Asparagus*, similar cases of lineage-specific transposon expansion have been found in the Asteraceae, where a small number of *Gypsy* families have been expanding since the branch leading to the Asteraceae (Staton and Burke 2015). We hypothesize that the proliferation of both *Gypsy* and *Copia* retroelements in dioecious lineages is associated with two coincident events in *Asparagus* evolution: range expansion and the origin of dioecy. As others have documented, range expansion out of South Africa is associated with a transition of ancestrally hermaphroditic *Asparagus* species to dioecy within a clade distributed across Europe and Asia. (Štajner *et al.* 2002; Kuhl *et al.* 2005; Kanno and Yokoyama 2011; Kubota *et al.* 2012; Norup *et al.* 2015). Founder populations formed during this range expansion with small effective population sizes may have been especially susceptible to weakly deleterious transposon proliferation due to the reduced strength of purifying selection relative to populations with large effective sizes (Lynch *et al.* 2011). In addition, the origin of sex chromosomes alone may have promoted proliferation of retrotransposons. Suppressed recombination within the region of the sex chromosomes where gender determination genes reside in the first dioecious *Asparagus* species may have harbored active retrotransposons. An excess of repetitive elements can be found in the nonrecombining regions of several plant Y chromosomes. For instance, the Y chromosomes in both *Silene* and papaya can be replete with, or entirely lacking, tandem arrays and LTR retroelements that distinguish them from both the X and other autosomes (Pritham *et al.* 2003; Filatov *et al.* 2009; VanBuren and Ming 2013). Recombination is selected against in these sex determination regions of a sex chromosome, given that recombination could break apart genes influencing male and female function, leading to the formation of neuters. As a consequence of nonrecombination, portions of Y (or W) chromosomes are particularly susceptible to the effects of Müller’s ratchet—an evolutionary process leading to the accumulation of slightly deleterious elements that can be accelerated in small effective population sizes (Charlesworth and Langley 1989; Moran 1996). Further, ectopic exchange (recombination at nonhomologous sites) is also predicted to control the proliferation of transposable elements in genomes by creating deleterious chromosomal rearrangements. While selected against in recombining portions of the genome, the rate of fixed ectopic exchange in nonrecombining regions of the Y is expected to be lower or zero, potentially leading to the proliferation of TE copy number through relaxed selection (Charlesworth and Langley 1989). This selection on young sex chromosomes may drive the maintenance and proliferation of repetitive elements, and, in concert with faster mutation rates and background selection, may lead to the initial expansion and subsequent degeneration of sex chromosomes (Engelstädter 2008). While the accumulation of repetitive elements on sex chromosomes has been well studied, understanding the spread of these elements to autosomes, and the subsequent contributions to genome size increase, will require additional comparative genomic analyses.

## Supplementary Material

Supplemental Material

## References

[bib1] Ainsworth C., 2000 Boys and girls come out to play: the molecular biology of dioecious plants. Ann. Bot. (Lond.) 86: 211–221.

[bib2] Akagi T. Henry I. M. Tao R. Comai L., 2014 A Y-chromosome-encoded small RNA acts as a sex determinant in persimmons. Science 346: 646–650. 10.1126/science.1257225 25359977

[bib3] Ashman T.L., A. Kwok, and B. C. Husband, 2013 Revisiting the dioecy-polyploidy association: alternate pathways and research opportunities. Cytogenet. Genome Res. 140: 241–255. 23838528 10.1159/000353306

[bib4] Bachtrog D., 2013 Y-chromosome evolution: emerging insights into processes of Y-chromosome degeneration. Nat. Rev. Genet. 14: 113–124. 23329112 10.1038/nrg3366 PMC4120474

[bib5] Bachtrog D. Hom E. Wong K. M. Maside X. de Jong P., 2008 Genomic degradation of a young Y chromosome in *Drosophila miranda*. Genome Biol. 9: R30. 18269752 10.1186/gb-2008-9-2-r30 PMC2374705

[bib6] Blanc G. Wolfe K. H., 2004 Functional divergence of duplicated genes formed by polyploidy during *Arabidopsis* evolution. Plant Cell 16: 1679–1691. 15208398 10.1105/tpc.021410 PMC514153

[bib7] Blondel V. D. Guillaume J. Lambiotte R. Lefebvre E., 2008 Fast unfolding of communities in large networks. J. Stat. Mech. Theory Exp. 2008: P10008.

[bib8] Boualem A. Fergany M. Fernandez R. Troadec C. Martin A., 2008 A conserved mutation in an ethylene biosynthesis enzyme leads to andromonoecy in melons. Science 321: 836–838. 18687965 10.1126/science.1159023

[bib9] Charlesworth B. Charlesworth D., 1978 A model for the evolution of dioecy and gynodioecy. Am. Nat. 112: 975–997.

[bib10] Charlesworth, B., and C. H. Langley, 1989 Population genetics of transposable elements in Drosophila, pp. 150–176 in *Evolution at the molecular level*, edited by R. Selander, A. Clark, and T. Whittam. Sinauer, Sunderland, MA.

[bib11] Cui L. Wall P. K. Leebens-Mack J. H. Lindsay B. G. Soltis D. E., 2006 Widespread genome duplications throughout the history of flowering plants. Genome Res. 16: 738–749. 16702410 10.1101/gr.4825606 PMC1479859

[bib12] Deng C.-L. L. Qin R.-Y. Y. Wang N.-N. N. Cao Y. Gao J., 2012 Karyotype of *Asparagus* by physical mapping of 45S and 5S rDNA by FISH. J. Genet. 91: 209–212. 22942092 10.1007/s12041-012-0159-1

[bib13] Dodsworth S. Chase M. W. Kelly L. J. Leitch I. J. Macas J., 2014 Genomic repeat abundances contain phylogenetic signal. Syst. Biol. 64: 112–126. 25261464 10.1093/sysbio/syu080 PMC4265144

[bib14] Doyle J. J. Doyle J. L., 1987 A rapid DNA isolation procedure for small quantities of fresh leaf tissue. Phytochem. Bull. 19: 11–15.

[bib15] Doyle J. J. Egan A. N., 2010 Dating the origins of polyploidy events. New Phytol. 186: 73–85. 20028472 10.1111/j.1469-8137.2009.03118.x

[bib16] Engelstädter J., 2008 Muller’s ratchet and the degeneration of Y chromosomes: a simulation study. Genetics 180: 957–967. 18780738 10.1534/genetics.108.092379 PMC2567394

[bib17] Filatov D. A., E. C. Howell, C. Groutides, and S. J. Armstrong, 2009 Recent spread of a retrotransposon in the *Silene latifolia* genome, apart from the Y chromosome. Genetics 181: 811–817. 19064703 10.1534/genetics.108.099267 PMC2644968

[bib46] Fukuda T. Ashizawa H. Suzuki R. Ochiai T. Nakamura T., 2005 Molecular phylogeny of the genus *Asparagus* (Asparagaceae) inferred from plastid petB intron and petD–rpoA intergenic spacer sequences. Plant Spec. Biol. 20: 121–132.

[bib47] Galbraith D. W. Lambert G. M. Macas J. Doležel J., 1998 Analysis of nuclear DNA content and ploidy in higher plants, pp. 7.6.1–7.6.21 in Current protocols in cytometry, edited by J. P. Robinson, Z. Darzynkiewicz, P. N. Dean, L. G. Dressler, A. Orfao *et al* John Wiley & Sons, New York. 10.1002/0471142956.cy0706s02 18770733

[bib18] Harkess A. Mercati F. Shan H. Y. Sunseri F. Falavigna A., 2015 Sex-biased gene expression in dioecious garden asparagus (*Asparagus officinalis*). New Phytol. 207: 883–892. 25817071 10.1111/nph.13389

[bib19] Hertweck K. L., 2013 Assembly and comparative analysis of transposable elements from low coverage genomic sequence data in Asparagales. Genome 56: 487–494. 24168669 10.1139/gen-2013-0042

[bib20] Hough J. Hollister J. D. Wang W. Barrett S. C. H. Wright S. I., 2014 Genetic degeneration of old and young Y chromosomes in the flowering plant *Rumex hastatulus*. Proc. Natl. Acad. Sci. USA 2014: 1–6. 10.1073/pnas.1319227111 PMC4040613 24825885

[bib21] Huang X. Madan A., 1999 CAP3: A DNA sequence assembly program. Genome Res. 9: 868–877. 10508846 10.1101/gr.9.9.868 PMC310812

[bib22] Kanno, A., and J. Yokoyama, 2011 Asparagus, pp. 23–42 in *Wild Crop Relatives: Genomic and Breeding Resources*, edited by C. Kole. Springer, Berlin.

[bib23] Kidwell M. G., 2002 Transposable elements and the evolution of genome size in eukaryotes. Genetica 115: 49–63. 12188048 10.1023/a:1016072014259

[bib24] Krähenbühl M. Yuan Y. M. Küpfer P., 2002 Chromosome and breeding system evolution of the genus *Mercurialis* (Euphorbiaceae): implications of ITS molecular phylogeny. Plant Syst. Evol. 234: 155–169.

[bib25] Kubota S. Konno I. Kanno A., 2012 Molecular phylogeny of the genus *Asparagus* (Asparagaceae) explains interspecific crossability between the garden asparagus (*A. officinalis*) and other *Asparagus* species. Theor. Appl. Genet. 124: 345–354. 21947345 10.1007/s00122-011-1709-2

[bib26] Kuhl J. C. Havey M. J. Martin W. J. Cheung F. Yuan Q., 2005 Comparative genomic analyses in *Asparagus*. Genome 1060: 1052–1060. 16391674 10.1139/g05-073

[bib27] Leitch I. J. Beaulieu J. M. Chase M. W. Leitch A. R. Fay M. F., 2010 Genome size dynamics and evolution in monocots. J. Bot. 2010: 1–18.

[bib28] Li S. F. Gao W. J. Zhao X. P. Dong T. Y. Deng C. L., 2014 Analysis of transposable elements in the genome of *Asparagus officinalis* from high coverage sequence data. PLoS One 9: 1–8. 10.1371/journal.pone.0097189 PMC4014616 24810432

[bib29] Lynch M. Bobay L.-M. Catania F. Gout J.-F. Rho M., 2011 The repatterning of eukaryotic genomes by random genetic drift. Annu. Rev. Genomics Hum. Genet. 12: 347–366. 21756106 10.1146/annurev-genom-082410-101412 PMC4519033

[bib30] Ma J. Bennetzen J. L., 2004 Rapid recent growth and divergence of rice nuclear genomes. Proc. Natl. Acad. Sci. USA 101: 12404–12410. 15240870 10.1073/pnas.0403715101 PMC515075

[bib31] McKain M. R. Wickett N. Zhang Y. Ayyampalayam S. McCombie W. R., 2012 Phylogenomic analysis of transcriptome data elucidates co-occurrence of a paleopolyploid event and the origin of bimodal karyotypes in *Agavoideae* (Asparagaceae). Am. J. Bot. 99: 397–406. 22301890 10.3732/ajb.1100537

[bib32] Ming R. Bendahmane A. Renner S. S., 2011 Sex chromosomes in land plants. Annu. Rev. Plant Biol. 62: 485–514. 21526970 10.1146/annurev-arplant-042110-103914

[bib33] Moran N. A., 1996 Accelerated evolution and Muller’s rachet in endosymbiotic bacteria. Proc. Natl. Acad. Sci. USA 93: 2873–2878. 8610134 10.1073/pnas.93.7.2873 PMC39726

[bib34] Norup M. F. Petersen G. Burrows S. Bouchenak-Khelladi Y. Leebens-Mack J., 2015 Evolution of *Asparagus* L. (Asparagaceae): Out-of-South-Africa and multiple origins of sexual dimorphism. Mol. Phylogenet. Evol. 92: 25–44. 26079131 10.1016/j.ympev.2015.06.002

[bib35] Pritham E. J. E. Zhang Y. H. Feschotte C. Kesseli R. R. V., 2003 An Ac-like transposable element family with transcriptionally active Y-linked copies in the white campion, *Silene latifolia*. Genetics 165: 799–807. 14573489 10.1093/genetics/165.2.799 PMC1462803

[bib36] Rousseau-Gueutin M. Gaston A., A. Aïnouche, M. L. Aïnouche,, K. Olbricht *et al*, 2009 Tracking the evolutionary history of polyploidy in *Fragaria* L. (strawberry): New insights from phylogenetic analyses of low-copy nuclear genes. Mol. Phylogenet. Evol. 51: 515–530. 19166953 10.1016/j.ympev.2008.12.024

[bib37] Štajner N. Bohanec B. Javornik B., 2002 Genetic variability of economically important *Asparagus* species as revealed by genome size analysis and rDNA ITS polymorphisms. Plant Sci. 162: 931–937.

[bib38] Staton S. E. Burke J. M., 2015 Evolutionary transitions in the *Asteraceae* coincide with marked shifts in transposable element abundance. BMC Genomics 16: 623. 26290182 10.1186/s12864-015-1830-8 PMC4546089

[bib39] Steinemann M. Steinemann S., 1998 Enigma of Y chromosome degeneration: neo-Y and neo-X chromosomes of *Drosophila miranda* a model for sex chromosome evolution. Genetica 102–103: 409–420. 9720292

[bib40] Suyama M. Torrents D. Bork P., 2006 PAL2NAL: Robust conversion of protein sequence alignments into the corresponding codon alignments. Nucleic Acids Res. 34(Web Server issue): W609–W612. 10.1093/nar/gkl315 PMC1538804 16845082

[bib41] Telgmann-Rauber A. Jamsari A. Kinney M. S. Pires J. C. Jung C., 2007 Genetic and physical maps around the sex-determining M-locus of the dioecious plant *Asparagus*. Mol. Genet. Genomics 278: 221–234. 17609979 10.1007/s00438-007-0235-z

[bib42] VanBuren R. Ming R., 2013 Dynamic transposable element accumulation in the nascent sex chromosomes of papaya. Mob. Genet. Elements 3: e23462. 10.4161/mge.23462 PMC3661139 23734293

[bib43] Vitte C. Estep M. C. Leebens-Mack J. Bennetzen J. L., 2013 Young, intact and nested retrotransposons are abundant in the onion and *Asparagus* genomes. Ann. Bot. (Lond.) 112: 881–889. 10.1093/aob/mct155 PMC3747808 23887091

[bib44] Wicker T. Sabot F. Hua-Van A. Bennetzen J. L. Capy P., 2007 A unified classification system for eukaryotic transposable elements. Natl. Rev. 8: 973–982. 10.1038/nrg2165 17984973

[bib45] Yang Z., 2007 PAML 4: Phylogenetic analysis by maximum likelihood. Mol. Biol. Evol. 24: 1586–1591. 17483113 10.1093/molbev/msm088

