# Supplementary material for: Retrotransposon Proliferation Coincident with the Evolution of Dioecy in Asparagus
Source: G3 (Bethesda). 2016 Jun 23;6(9):2679–85. doi: 10.1534/g3.116.030239 (PMC5015926; doi:10.1534/g3.116.030239)
Supplement: Supplemental Material [file supp_6_9_2679__index.html]

Retrotransposon Proliferation Coincident with the Evolution of Dioecy in Asparagus — Supplemental Material 

# Retrotransposon Proliferation Coincident with the Evolution of Dioecy in *Asparagus*

## Supplemental Material for Harkess *et al.*, 2016

**Files in this Data Supplement:**

- Table S1 - Transcriptome assembly and translation. (.pdf, 223 KB)
- Table S2 - Number of paralogous and orthologous transcript pairs analyzed. (.pdf, 83 KB)
- Table S3 - Estimated shotgun sequencing genome coverage. (.pdf, 118 KB)
