# Supplementary material for: Retrotransposon Proliferation Coincident with the Evolution of Dioecy in Asparagus
Source: G3 (Bethesda). 2016 Jun 23;6(9):2679–85. doi: 10.1534/g3.116.030239 (PMC5015926; doi:10.1534/g3.116.030239)
Supplement: Supplemental Material [file supp_g3.116.030239_TableS1.pdf]

Table S1: Transcriptome assembly and translation

Species	Sexual System	Number of Transcripts	Number of translated transcripts	Translated cDNA N50 (nt)
A. asparagoides	Hermaphrodite	118,517	40,928	1,431
A. officinalis	Dioecious	276,556	158,386	1,482
