# Supplementary material for: Retrotransposon Proliferation Coincident with the Evolution of Dioecy in Asparagus
Source: G3 (Bethesda). 2016 Jun 23;6(9):2679–85. doi: 10.1534/g3.116.030239 (PMC5015926; doi:10.1534/g3.116.030239)
Supplement: Supplemental Material [file supp_g3.116.030239_TableS2.pdf]

Table S2: Number of paralogous and orthologous transcript pairs analyzed

Comparison	Pairs analyzed
A. officinalis x A. officinalis	225,035
A. asparagoides x A. asparagoides	11,818
A. officinalis x A. asparagoides	326,687
