# Supplementary material for: Retrotransposon Proliferation Coincident with the Evolution of Dioecy in Asparagus
Source: G3 (Bethesda). 2016 Jun 23;6(9):2679–85. doi: 10.1534/g3.116.030239 (PMC5015926; doi:10.1534/g3.116.030239)
Supplement: Supplemental Material [file supp_g3.116.030239_TableS3.pdf]

Table S3: Estimated shotgun sequencing genome coverage

Species	Sequence data (nt)	Genome size (nt)	Estimated coverage
A. officinalis	7,955,329	1,370,000,000	0.0058
A. maritimus	14,586,484	1,310,000,000	0.0111
A. aphyllus	13,805,190	1,250,000,000	0.0110
A. stipularis	9,569,792	1,090,000,000	0.0089
A. falcatus	8,238,184	1,060,000,000	0.0078
A. virgatus	13,374,059	830,000,000	0.0161
A. pyrimidalis	16,872,384	720,000,000	0.0234
A. asparagoides	12,304,847	2,400,000,000	0.0051
